# Myeloid-associated differentiation marker is associated with type 2 asthma and is upregulated by human rhinovirus infection

**DOI:** 10.3389/fimmu.2023.1237683

**Published:** 2023-08-11

**Authors:** Sasipa Tanyaratsrisakul, Alane Blythe C. Dy, Francesca Polverino, Mari Numata, Julie G. Ledford

**Affiliations:** ^1^ Asthma and Airway Disease Research Center, University of Arizona, Tucson, AZ, United States; ^2^ Department of Medicine, Baylor College of Medicine, Houston, TX, United States; ^3^ Department of Medicine, National Jewish Health, Denver, CO, United States; ^4^ Department of Cellular and Molecular Medicine, University of Arizona, Tucson, AZ, United States

**Keywords:** MYADM, asthma, human rhinovirus, airway epithelial cells, lung inflammation

## Abstract

**Background:**

Human rhinoviruses are known to predispose infants to asthma development during childhood and are often associated with exacerbations in asthma patients. MYADM epithelial expression has been shown to associate with asthma severity. The goal of this study was to determine if MYADM expression patterns were altered in asthma and/or rhinovirus infection and if increased MYADM expression is associated with increased asthma-associated factors.

**Methods:**

Utilizing H1HeLa cells and differentiated primary human airway epithelial cells (AECs), we measured the expression of MYADM and inflammatory genes by qRT-PCR in the presence or absence of RV-1B infection or poly I:C treatment and with siRNA knockdown of MYADM. Expression of MYADM in the asthmatic lung was determined in the ovalbumin (ova)-challenged murine model.

**Results:**

MYADM expression was upregulated in the lungs from ova-treated mice and in particular on the subsurface vesicle membrane in airway epithelial cells. Upon infection with RV-1B, human AECs grown at an air–liquid interface had increased the MYADM expression predominantly detected in ciliated cells. We found that the presence of MYADM was required for expression of several inflammatory genes both in a resting state and after RV-1B or poly I:C treatments.

**Conclusions:**

Our studies show that in a mouse model of asthma and during RV-1B infection of primary human AECs, increased MYADM expression is observed. In the mouse model of asthma, MYADM expression was predominantly on the luminal side of airway epithelial cells. Additionally, MYADM expression was strongly associated with increases in inflammatory genes, which may contribute to more severe asthma and RV-linked asthma exacerbations.

## Introduction

Myeloid-associated differentiation marker (MYADM) is a transmembrane protein that has been identified as a marker of hematopoietic progenitor cells and is upregulated during myeloid differentiation (1). While MYADM was first discovered in hematopoietic progenitor cells, additional studies have noted MYADM gene expression in human spleen, lung, liver, kidney, testis, prostate, skeletal muscle, ovary, and peripheral blood leukocytes (2). Several mechanisms have been described, which indicate that MYADM influences cell physiology and is involved in the pathogenesis of diseases such as cancer (3–6) and hypertension (7–10). MYADM is also involved in cell membrane organization for cell spreading and migration by recruiting Rac1 to the membrane raft in HeLa cells (11). Knockdown of MYADM in primary endothelial cells impaired cell–cell junction formation, increased ICAM-1 expression, and promoted leukocyte adhesion (12).

Our group recently reported that blocking the activity of MYADM in a murine asthma model disrupts eosinophils clearance, increases airway hyperresponsiveness, and results in increased mucus production (13). We also discovered that among asthmatic patients, MYADM expression is markedly increased in human airway epithelial cells and MYADM levels are associated with more severe asthma. Moreover, increased MYADM expression in airway epithelial cells also associated with increased peripheral blood eosinophilia and exhaled nitric oxide, as well as frequency of exacerbation (13).

Despite optimal medical therapy, many asthma patients still experience exacerbations, which is an increase in existing inflammatory processes that worsens airway obstruction (14). The most frequent trigger of exacerbation is infectious respiratory illness (14, 15). Although infection with rhinoviruses (RVs) typically result in only mild symptoms in healthy individuals without underlining pulmonary diseases, RVs have been shown to induce wheezing early in life, with subsequent development of asthma in some individuals (15–18). Moreover, RV exposure frequently induces airway hyperresponsiveness, which can lead to exacerbations of asthma and fixed airflow limitation in both children and adults (18–23).

In this study, we sought to determine if viral infection induces MYADM expression, which based on our previous findings would be associated with enhanced inflammatory responses in airway epithelial cells. We discovered that Rhinovirus A (RV-1B), and not RSV, infection led to increases of MYADM expression in both H1Hela cells and differentiated primary bronchial epithelial cells (AEC). Furthermore, siRNA knockdown of MYADM did not affect viral replication but reduced inflammatory cytokine production in the cells during both resting state and during challenges with either poly I:C or RV-1B.

## Results

### Human rhinovirus-1B infection upregulates MYADM expression in epithelial cells

**Figure 1:**
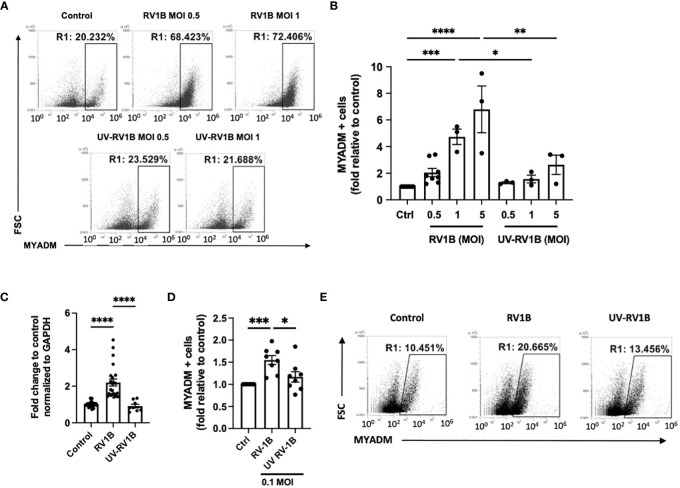
Human rhinovirus 1B infection increased the MYADM expression in H1HeLa and in primary airway epithelial cells. **(A–C)** H1HeLa cells were infected with RV1B or UV-RV1B at MOI of 0.5, 1, or 5 for 48 h Cells were stained with the anti-MYADM antibody followed by anti-rabbit IgG conjugated with Alexa Fluor 488. **(A)** Dot plots representative of the flow cytometry assay and **(B)** the number of cells harboring MYADM are shown as fold relative to control. **(C)** MYADM transcription determined by qRT-PCR at 48 h after infection at MOI of 0.1 in H1HeLa cells. **(D)** Primary human AECs were grown at ALI and infected with RV1B or UV-RV1B at MOI of 0.1 for 48 h The numbers of MYADM-positive cells were detected by flow cytometry and present as fold relative to control. **(E)** Representative data from the flow cytometry assay of AECs as described for **(D)** The mean± SEM from three independent experiments were plotted in the graphs. Results were analyzed using one-way ANOVA and Tukey’s multiple comparisons test in Prism software. *P < 0.05; **P, 0.01; ***P < 0.001; ****P < 0.0001.

To determine the effects of human rhinovirus infection on MYADM expression in epithelial cells, we first infected H1HeLa cells with rhinovirus-1B (RV-1B) and quantified MYADM+ cells by flow cytometry assay (**Figures 1A, B**). At the 0.5 multiplicity of infection (MOI), cells expressing MYADM increased twofold compared with those of non-infected controls. The increased expression was dose-dependent, with 4.7- and 6.8-fold increases at MOI of 1 and 5, respectively. MYADM upregulation was also replication-dependent as UV-irradiated RV-1B (UV-RV1B) did not increase MYADM-expressing cells.

MYADM gene expression in H1HeLa cells was further quantified by qRT-PCR (**Figure 1C**). At MOI 0.1, MYADM mRNA levels increased 2.2-fold compared with the control. MYADM mRNA levels were not increased with UV-RV1B challenge. Moreover, MYADM expression in differentiated human primary epithelial cells (AECs) at air–liquid interface (ALI) cultures also increased upon RV-1B infection (**Figures 1D, E**). Interestingly, another common respiratory virus, RSV, did not induce MYADM expression (**Supplement Figure 1**).

### MYADM expression is increased in lung epithelial cells from asthmatic mice

**Figure 2:**
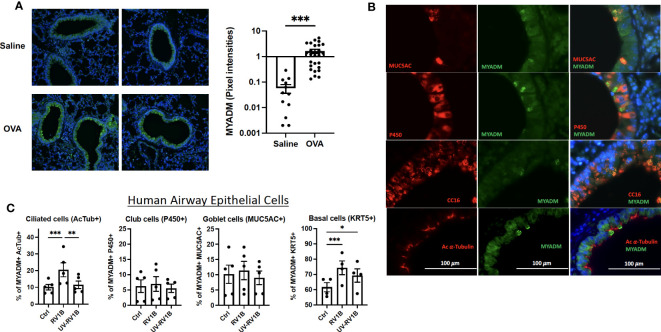
Lungs from mice with allergic airways disease had increased MYADM expression on differentiated epithelial cells and RV-1B-increased ciliated cells in human AECs. Lung slides of control or OVA-treated mice were stained with cell markers, acetylated α-Tubulin, cytochrome P450, CC16, or MUC5AC in red; MYADM in green; and nucleus with DAPI in blue. The airway images were taken using Axiophot (Carl Zeiss Microimaging) **(A, B)** or Leica DM1600 **(B)**, 10 images per lung slide, three slides/mice, and five to six mice per group. The intensity of MYADM in green was determined using ImageJ software; each dot represents collective intensity per slide **(A)**. Two representative images of airways are shown for each condition. Mean± SEM is shown and analyzed using unpaired t test from Prism software. **(C)** Differentiated human AECs were infected with RV1B or UV-RV1B at MOI of 0.1 for 48 h Cells were detected with acetylated α-Tubulin, cytochrome P450 (P450), MUC5AC, or Keratin 5 (KRT5) antibody followed by anti-mouse IgG conjugated with Alexa fluor 647 by flow cytometry. Mean± SEM of cell count by the flow cytometry assay from five independent experiments is shown. The results were analyzed by two-way ANOVA using Prism software. *P < 0.05; **P < 0.01; ***P < 0.001.

We next examined MYADM expression in lung sections from a murine model of allergic airways disease. As shown in **Figure 2A**, the MYADM protein level (shown in green) in ovalbumin-treated lung sections from mice was increased ~25-fold compared with the saline controls. MYADM appeared to localize to the membrane of cytoplasmic vesicles, which were apically polarized toward the airway’s lumen (**Figure 2B**). In mouse airways, MYADM was mainly colocalized with cells staining positive for CC16 (club cells) and MUC5AC (goblet cells), whereas some staining was also detected in ciliated cells.

In human AECs, RV-1B infection led to increases in total ciliated and basal cell numbers (**Figure 2C**), as determined by flow cytometry, whereas the percentage of MYADM-expressing cells for each respective cell type remained consistent (**Supplement Figure 2**).

### Downregulation of MYADM leads to decreased cytokine/chemokine production

**Figure 3:**
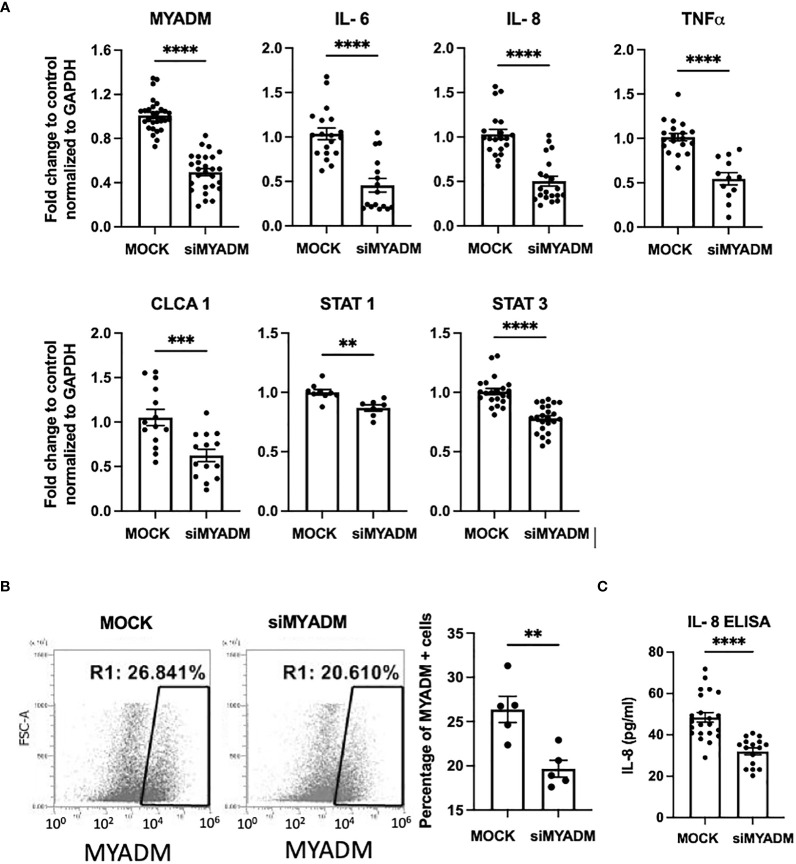
Knockdown of MYADM reduced inflammatory cytokine expression in H1HeLa cells. H1HeLa cells were treated with 50 nM MYADM siRNA or scramble siRNA (MOCK) control in 1 µg/ml Lipofectamine 2000. Cells were harvested for flow cytometry and RNA extraction at 72 h after transfection. **(A)** qRT-PCR for MYADM, IL-6, IL-8, TNFα, CLCA1, and STAT3 normalized with GAPDH presented as mean± SEM of fold change over MOCK control. **(B)** The representative dot plot from the flow cytometry assay and graph representing mean± SEM from five individual experiments. **(C)** IL-8 concentrations in culture media were measured by ELISA. The mean± SEM from three independent experiments. Results were analyzed using unpaired t-test by Prism software. **P < 0.01; ***P < 0.001; ****P < 0.0001.

To examine the role of MYADM in inflammation, we performed siRNA knockdown of MYADM in H1HeLa cells. As shown in **Figure 3A**, knockdown of MYADM reduced MYADM transcription by half (0.49-fold). The transcription levels of inflammatory cytokines, IL-6, IL-8, and TNFα, were also reduced by half (0.46, 0.50, and 0.54) in cells in which MYADM was knocked down, compared with controls. Transcription of calcium-activated chloride channel regulator 1 (CLCA1) and signal transducer and activator of transcription (STAT)-1 and -3 were also reduced to 0.62, 0.86, and 0.78, respectively. Additionally, cells expressing MYADM were quantified by flow cytometry (**Figure 3B**). The percentage of cells carrying MYADM was 26% for mock control (scramble siRNA) and 20% for the siRNA-treated cells. While MYADM knockdown at the protein levels was only modest, it still resulted in a significant decrease of IL-8 secretion into culture media compared with controls (**Figure 3C**).

### MYADM expression is associated with RV-1B replication and RV-1B-induced responses in epithelial cells

We next sought to determine whether the induction of MYADM expression is associated with RV-1B replication by correlation analysis of gene transcripts (**Supplementary Figure 3**). Increased MYADM expression significantly collerated with increased RV-1B replication in H1HeLa cells (**Supplementary Figure 3A**) and in AECs (**Supplementary Figure 3B**). For H1HeLa cells, several RV-1B-induced genes had significant positive associations with MYADM expression, including interferon-lambda (IFN-l), IL-8, STAT-3, and CLCA1. In differentiated AECs, the detectable RV-1B-induced genes that had significant positive association with MYADM expression included the IFN-related genes IFN-l, interferon-stimulated gene 15 (ISG15), ubiquitin-specific peptidase 18 (USP18), IFN-Λ receptor 1 (IFNΛ-R1), and IFN-β1; the cell receptors Toll-like receptor 3 (TLR3), low-density lipoprotein receptor (LDLR), ICAM-1, and melanoma differentiation-associated gene 5 (MDA5); and other inflammatory factors STAT1 and C–X–C motif chemokine ligand 11 (CXCL11). The correlation (R) of each gene transcript of interest to MYADM and the corresponding P-values are presented in **Table 1**.

**Table 1:** MYADM expression correlation with RV-1B replication and RV-1B-induced inflammatory responses.

*H1HeLa cells*		*IFNL*	*IL8*	*MDA5*	*STAT3*	*CLCA1*	*RV1B*	
*R*		0.630	0.441	0.261	0.473	0.586	0.847	
*P (two-tailed)*		0.000	0.027	0.155	0.015	0.008	<0.0001	
*AECs cells*	*IFNL*	*IL8*	*MDA5*	*STAT3*	*CLCA1*	*RV1B*	*STAT1*	*CXCL11*
*R*	0.669	0.500	0.587	0.376	0.770	0.729	0.634	0.599
*P (two-tailed)*	0.017	0.098	0.045	0.228	0.073	0.007	0.027	0.039
*AECs cells*	*ISG15*	*USP18*	*IFN β1*	*IFNR1*	*Col-1*	*LDLR*	*ICAM-1*	*TLR3*
*R*	0.622	0.648	0.632	0.856	0.547	0.843	0.893	0.623
*P (two-tailed)*	0.031	0.023	0.027	0.003	0.066	0.004	0.001	0.030

H1HeLa cells or differentiated AECs grown at ALI were infected with RV-1B at MOI 0.1 for 48 h. Transcription of target genes was quantified by qRT-PCR. The fold changes to control normalized to GAPDH from three independent experiments were plotted on the graph. The correlation of gene expression compared with MYADM was calculated using Pearson correlation coefficients in GraphPad Prism software.

### MYADM knockdown reduced the levels of RV-1B-induced STAT-3 and IL-8 in epithelial cells

**Figure 4:**
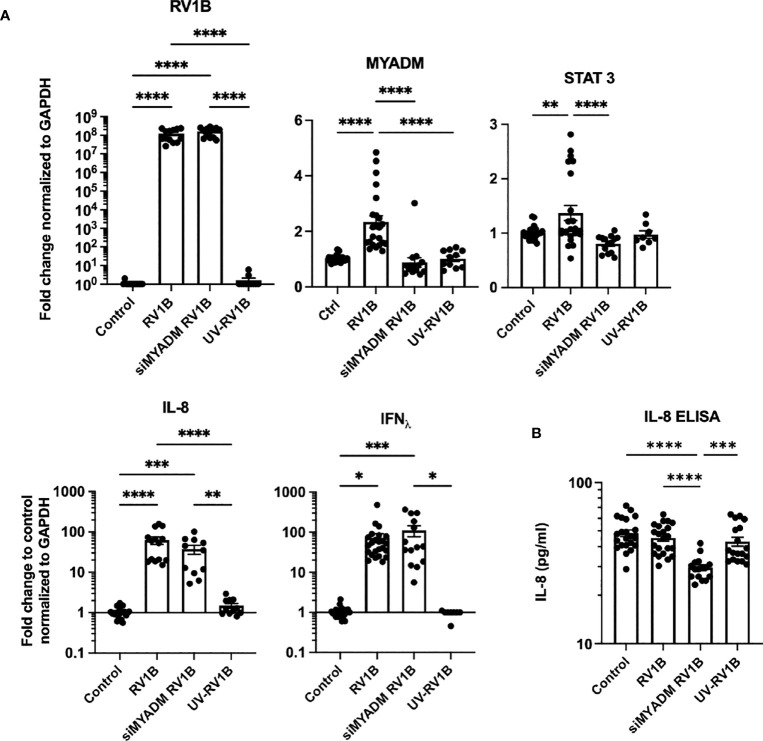
Knockdown of MYADM reduced RV1B-induced STAT3 transcription and IL-8 secretion. H1HeLa cells were infected with RV1B or UV-RV1B at MOI of 0.1 for 48 h. **(A)** The cycle threshold (C_T_) from qRT-PCR was normalized with GAPDH and present as fold changes over control. For RV1B detection, C_T_ was compared with the highest measurable C_T_ (C_T_ = 38). **(B)** IL-8 ELISA was performed using 1:20 dilution of culture media. The mean± SEM from three to four independent experiments are shown. Results were analyzed using one-way ANOVA and Tukey’s multiple comparisons test by Prism software. *P < 0.05; **P < 0.01; ***P < 0.001; ****P < 0.0001.

To further examine the role of MYADM during RV-1B infection, H1HeLa cells were transfected with MYADM siRNA followed by RV-1B infection. Quantitative RT-PCR revealed that both MYADM knockdown and control cells had comparable RV-1B replication (**Figure 4A**). The infection led to increased MYADM mRNA by 2.3-fold, whereas RV-1B infection was not able to induce further MYADM upregulation in MYADM-silenced cells. UV-RV1B-treated cells did not have a detectable change in MYADM expression, which were similar to controls. STAT3 was increased by RV-1B infection and was significantly decreased when MYADM was knocked down. IL-8 and IFN_Λ_ were increased significantly by RV1B infection; MYADM knockdown led to a slight, non-significant reduction in the induction. When assessing IL-8 protein levels, as shown in **Figure 4B**, whereas IL-8 protein concentrations in culture media were not altered by RV-1B infection, cells in which MYADM was knocked down during infection had a significant reduction in IL-8 protein secretion.

### Poly I:C increased MYADM *via* TLR3 activation and upregulation of IL-8, STAT3, and TLR are MYADM-dependent

**Figure 5:**
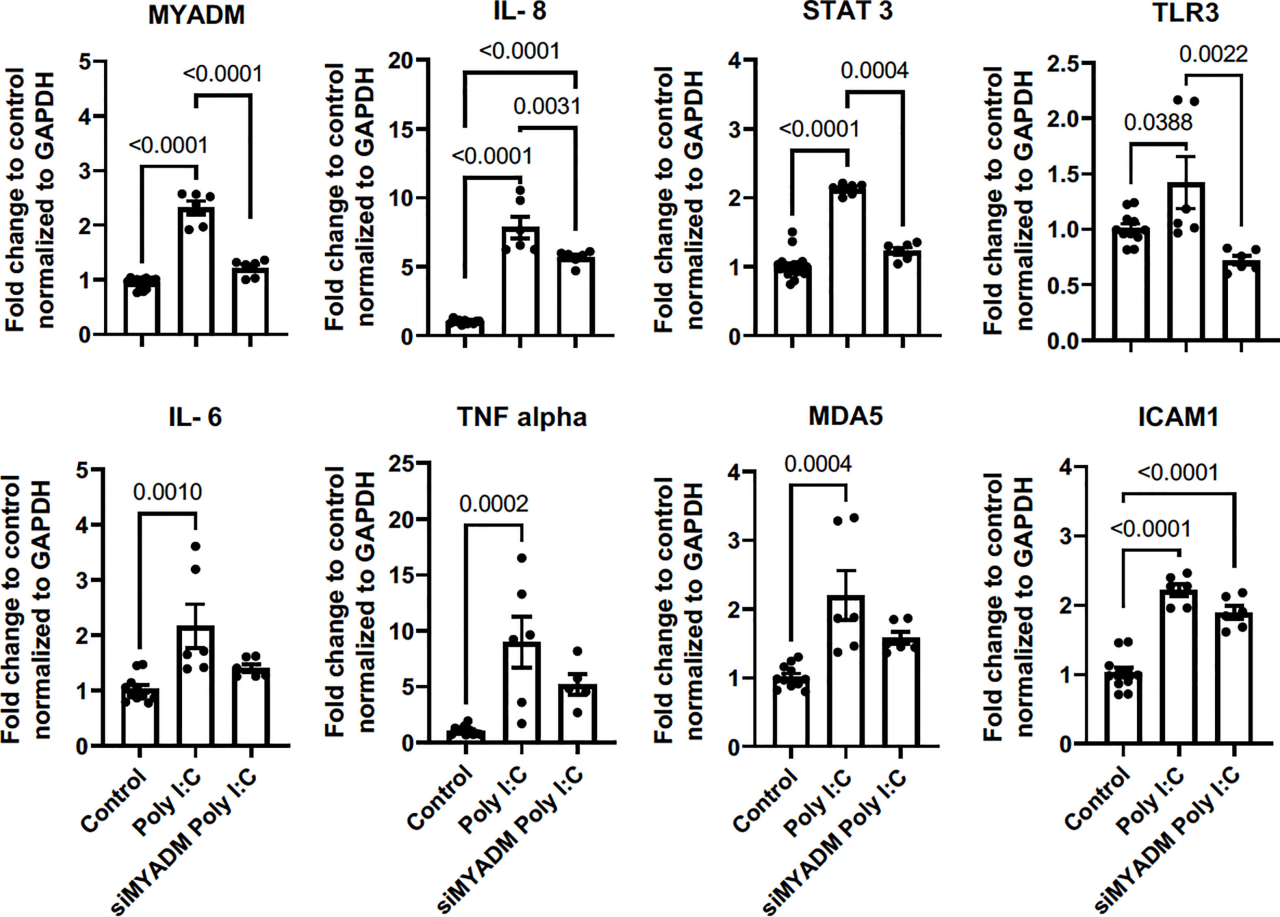
Poly I:C increased MYADM via TLR3 activation, and upregulation of IL-8, STAT3, and TLR is MYADM dependent. H1HeLa cells were treated with 10 μg/ml Poly I:C for 48 h. The cycle threshold (C_T_) from the qRT-PCR assay was normalized with GAPDH and presents as mean ± SEM of fold change over control from three experiments. The results were analyzed using one-way ANOVA and Tukey’s multiple comparisons test. Line and number display P values of selected comparisons.

We next assessed whether TLR3 stimulation increased MYADM expression as we observed with live RV1B infection. For this, H1HeLa cells were incubated with poly I:C for 48 h and RT-PCR was performed. As shown in **Figure 5**, poly I:C increased the transcription of MYADM, IL-6, IL-8, and TNFα, STAT3, TLR3, MDA5, and ICAM-1. Knockdown of MYADM led to a reduction in Poly I:C-induced expression of MYADM, IL-8, STAT-3, and TLR-3 but did not alter the expression of Poly I:C-induced IL-6, TNF-a, MDA5, or ICAM-1.

## Discussion

We had recently found that blocking of MYADM in a murine asthma model disrupts eosinophil clearance, leads to increased airway hyperresponsiveness, and results in increased mucus metaplasia in the airways (13). Moreover, MYADM expression in human airway epithelial cells was markedly increased in asthmatic patients compared with controls and its expression was positively associated with peripheral blood eosinophilia, exhaled nitric oxide, and the frequency of exacerbation (13). These findings strongly suggest that MYADM contributes to airways’ inflammatory responses in asthma. Our studies presented here add to the growing body of evidence for mechanisms associated with MYADM in the respiratory system as we are the first, to our knowledge, to determine the impact of rhinovirus infection on the expression of MYADM and the relevance of MYADM expression on RV-induced inflammatory responses.

In our attempt to learn more about MYADM and its connection to severe asthma, we chose to examine the response to Rhinovirus a virus known to be associated with asthma exacerbations. In fact, rhinovirus-induced wheezing early in life is associated with subsequent development of asthma and viral exposure is frequently associated with exacerbations of asthma in children and adults, as well as exacerbations of patients with COPD (15, 18, 19, 22, 23). Interestingly, we found that respiratory syncytial virus (RSV), another common cause of respiratory infection in children (16), did not induce MYADM expression in epithelial cells suggesting viral specificity to MYADM upregulation. This led us to the question what role MYADM may play in RV-induced inflammatory responses and whether regulation of MYADM may be a key factor leading to RV-induced exacerbations.

For our studies, we used a combination of HeLa cells and primary airway epithelial cells, which are the primary target of RV infection. The H1HeLa cell line is highly susceptible to RV1B infection and was grown in monolayers. In contrast, the differentiated primary airway epithelial cells were grown at an air–liquid interface and formed multilayers on collagen-coated Transwell inserts. Although the RV-induced gene profiling was slightly different, MYADM was upregulated in both systems. Cells expressing MYADM increased with increasing MOI, and MYADM transcription was dependent on viral replication. The replication-deficient, UV-irradiated RV-1B failed to increase MYADM in either cell type. These indicated increased MYADM expression in these studies varied directly to the level of RV-1B replication.

Enhanced MYADM expression in histological sections from mice exposed to an allergic airways challenge was also observed and was in line with our previous finding of increased MYADM expression in lung epithelial cells from asthma patients compared with healthy controls (13). From stained sections, MYADM appeared to be localized exclusively in airway epithelial cell cytoplasmic membranes and accumulated toward the airway lumen. This was in contrast to the expression of recombinant MYADM in COS cells and HEK293 cells, where the protein resided predominantly at the nuclear membrane and plasma membrane (2, 4), respectively. MYADM was detected in all four cell types examined from mouse lung sections—ciliated, goblet, club, and basal—and appeared to colocalize more with goblet and club cells. Interestingly, in human AECs grown at an ALI, RV-1B infection increased the number of basal cells and ciliated cells expressing MYADM, whereas MYADM-expressing club cells and goblet cells remained constant.

Further mechanistic questions remain to be answered in regard to how RV infection signals to the cells for MYADM to be upregulated. While RV-induced upregulation of MYADM was viral replication-dependent, we also observed an increased MYADM expression in lung from allergic mice and in cells stimulated with the viral mimic, Poly I:C, suggesting that there may be several modes by which MYADM expression is increased or that some common secondary downstream agent is involved. Further studies are warranted to better determine how MYADM expression is upregulated in the context of asthma and RV infection.

We observed significant reductions in several key inflammatory cytokines and effector proteins when MYADM was knocked down—whether in the context of infection or in naïve conditions. In the context to RV infection, MYADM expression was not only positively associated with RV-1B replication but also correlated positively with induced interferon responses, *IL-8*, *STAT1/3*, *CLCA1*, *CXCL11*, *TLR3*, *LDLR*, and *ICAM-1*. Interestingly, RV-1B infection in MYADM-silenced epithelial cells resulted in reduced *STAT3* expression and IL-8 secretion even though viral replication was similar. These results suggest that MYADM regulates RV-1B-induced inflammation but not RV-1B infection directly.

Our data are in line with others’ in that TLR3 is stimulated during RV-1B stimulation (24, 25). To rule out the requirement of MYADM for the responses of TLR3 activation, poly I:C was used as a viral mimic. To our surprise, these results indicated that MYADM was necessary for complete TLR3 responses. MYADM expression was also induced after poly I:C treatment in H1HeLa cells, mimicking what we see in RV-1B infection. Poly I:C induction of *IL-8*, *STAT3*, and *TLR3* was abolished when MYADM was knocked down. In contrast to RV-1B infection, *IFNΛ* and *CXCL11* were not induced by TLR3 activation alone. This suggested that TLR3 stimulation may be sufficient for MYADM induction during RV-1B infection. These data show that TLR3 activation increases MYADM expression and MYADM is required for upregulation of inflammatory cytokines IL-8, STAT3, and TLR3 itself, upon TLR3 activation.

**Figure 6:**
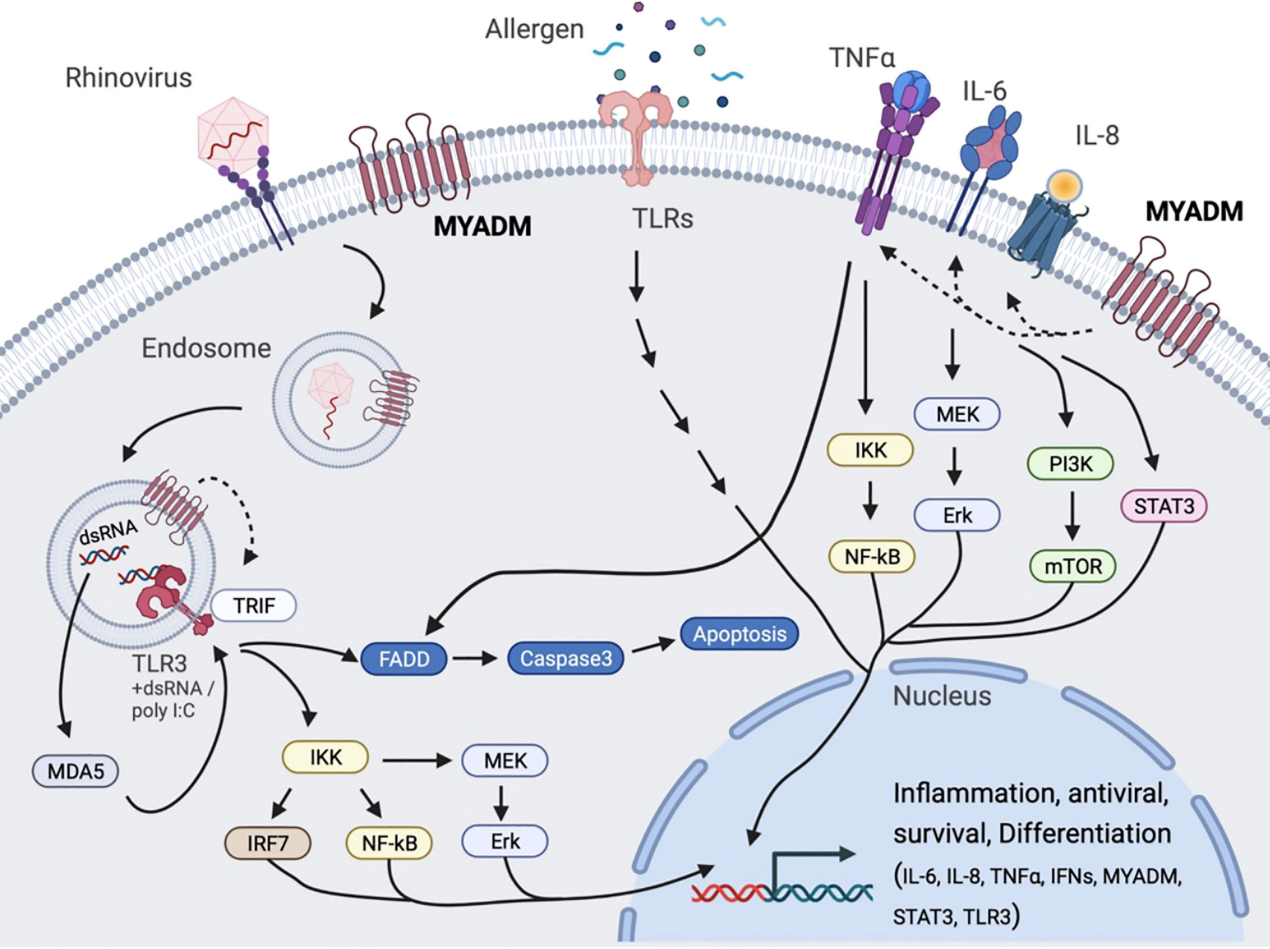
Proposed model for MYADM regulation of cytokines during allergic airway inflammation and rhinovirus infection. Airway allergens and double-stranded RNA produced during viral replication stimulate Toll-like receptors (TLRs) resulting in an increased expression of MYADM, as well as genes for inflammation, antiviral responses, survival, and differentiation. Increased MYADM expression on the cell surface is required for expression of IL-6, IL-8, TNFα, IFNs, CLCA1, STAT1/3, and TLR3 during the resting stage and/or stimulated stage.

Taken together, our data provide evidence that suggests that MYADM may contribute to the pathogenesis of asthma by mediating inflammatory responses in the lung. With MYADM expression increased in asthma and during acute RV infection, enhanced MYADM activity could therefore contribute to more pro-inflammatory cytokine signaling that could contribute to enhanced asthma severity, as well as asthma exacerbations. We propose a model in which MYADM regulates inflammatory cytokine production in a resting state and in both rhinovirus- and allergen-stimulated states (**Figure 6**). To our knowledge, this is the first report for MYADM expression in which it plays a role in airway inflammatory response in allergy and rhinovirus infections. Further studies are required to assure the application of MYADM in regulating inflammation and delve into its potential as a therapeutic target. Additionally, questions remain including how asthmatic airways maintain high MYADM levels, how MYADM signals influence inflammatory mediator expression, and if exacerbations could be prevented when MYADM is kept in balance.

## Methods

### Primary human bronchial airways epithelial cell isolation

Human AECs were isolated from discarded bronchial segments from donor lungs that were collected for transplant according to the protocol approved by University of Arizona’s Institutional Review Board (under PI, Dr. Polverino). Upon arrival in the lab, the bronchus was rinsed with RPMI1640 and submerged in 1 mg/ml protease (Sigma-Aldrich, St. Louis, MO) in Ham’s F12 for 1 h at 37°C. After neutralizing with HyClone FBS (Cytiva, Marlborough, MA), AECs were gently scrapped out and incubated in Gibco Versene (Thermo Fisher Scientific, Waltham, MA). AECs were washed and seeded on a 100 mm collagen and fibronectin-coated tissue culture dish in PneumaCult EX Plus supplemented with 10 µM ROCK inhibitor (APExBIO, Houston, TX) until 90% confluency. Cells were collected using trypsin and preserved in PneumaCult EX Plus (STEMCELL Technologies, Vancouver, BC) containing 30% FBS and 10% DMSO at 10^6^ cells/ml and stored at −80°C.

### Cell culture

The H1HeLa cell (CRL-1958) was purchased from ATCC and maintained in DMEM/F12 supplemented with 10% FBS and 1% penicillin–streptomycin (Thermo Fisher Scientific). Human AECs were cultured (26) in PneumaCult EX Plus until 80% confluency. Then, 2 × 10^4^ cells were seeded onto Transwells in a 24-well plate (Thermo Fisher Scientific) that was precoated with collagen and fibronectin. Once cells achieved 100% confluency, they were differentiated for 21 days with PneumaCult-ALI (STEMCELL Technologies, Vancouver, BC) media on a basolateral chamber only.

### Human rhinovirus-1B preparation

RV-1B (VR-1645) was obtained from ATCC propagated in a H1HeLa cell monolayer. First, after 1 h of viral attachment at MOI of 10, the infection was conducted in T25 flasks with DMEM/F12 10% NEAA at 33°C for 24 h. Then, the cell lysate supernatant was inoculated onto cells in T175 flasks. After 48 h, cells were lysed with 10% NP-40 (Sigma-Aldrich) and the supernatant was treated with RNase A (Thermo Fisher Scientific), lauryl sarcosine (Sigma-Aldrich), and β-mercaptoethanol. Next, the supernatant was overlaid on 30% sucrose and centrifuged at 40,000 RPM for 2 h. Finally, the particles were resuspended in 0.01%BSA Dulbecco’s phosphate-buffered saline (DPBS), filter-sterilized, and stored at −80°C until use. The purified RV1B were quantified by the plaque assay as described earlier (27) with some modification. First, the H1HeLa cell monolayers in the six-well plate were washed with DPBS with calcium and then incubated with various dilutions of RV-1B at room temperature for 1 h. The washed cells were subsequently overlaid with agarose and incubated at 33°C for 80 h. Plaques were fixed and stained with crystal violet. GFP-RSV was a generous gift of Drs. Mark Peeples and Peter Collins of the Ohio State University and propagated and used as previously detailed (28).

### MYADM knockdown

H1HeLa cells were cultured in a 24-well plate. Once cells were at 70% confluency, they were treated with 50 nM MYADM siRNA (Integrated DNA Technologies, Coralville, Iowa, **Table 2**), or scramble siRNA control in 1 µg/ml Lipofectamine 2000 (Thermo Fisher Scientific) according to the manufacturer’s instructions. Cells were harvested for RNA extraction or flow cytometry at 72 h after transfection.

**Table 2:** siRNA, qRT-PCR probes, and primer sequences.

Target	Sequence (5′–3′) or reference
siRNA MYADM (12)	GGUCUAAGACUCUCCCAAG
GAPDH TaqMan probe	Hs02786624_g1
MYADM TaqMan probe	Hs01091180_M1
RV1B F	CCTCCGGCCCCTGAAT
RV1B R	AAACACGGACACCCAAAGTAGT
IL-6 F	GACCCAACCACAAATGCCA
IL-6 R	GTCATGTCCTGCAGCCACTG
IL-8 F	CTGGCCGTGGCTCTCTTG
IL-8 R	CCTTGGCAAAACTGCACCTT
TNFα F	CTCTTCTGCCTGCTGCACTT
TNFα R	GGCTACAGGCTTGTCACTC
CLCA1 F	ATGGCTATGAAGGCATTGTCG
CLCA1 R	TGGCACATTGGGGTCGATTG
STAT1 F	TCCCCAGGCCCTTGTTG
STAT1 R	CAAGCTGCTGAAGTTCGTACC
STAT3 F	CTTTGAGACCGAGGTGTATCACC
STAT3 R	GGTCAGCATGTTGTACCACAGG
IFNΛ F	GCCAAAGATGCCTTAGAAGAG
IFNΛ R	CAGAACCTTCAGCGTCAGG
IFNβ F	GAACCTCCTGGCTAATGTCTATC
IFNβ R	TCCTTGGCCTTCAGGTAATG
IFNΛ R1 F	CAGTGTCCCGAAATACAGCA
IFNΛ R1 R	TGTGTCCAGAAAAGTCCAGGGC
CXCL11 F	AAGGACAACGATGCCTAAATCCC
CXCL11 R	CAGATGCTCTTTTCCAGGACTTC
TLR3 F	TCACTTGCTCATTCTCCCTTAC
TLR3 R	CTGTGAGTTCTTGCCCAATTTC
MDA5 F	GAAGTACAATGAGGCCCTACAA
MDA5 R	CATCACCACCCTCATCACTATC
ICAM I F	AGCGGCTGACGTGTGCAGTAAT
ICAM I R	TCTGAGACCTCTGGCTTCGTCA
LDLR F	GAATCTACTGGTCTGACCTGTCC
LDLR R	GGTCCAGTAGATGTTGCTGTGG
ISG15 F	TCCTGCTGGTGGTGGACAA
ISG15 R	TTGTTATTCCTCACCAGGATGCT
USP18 F	CTCAGTCCCGACGTGGAACT
USP18 R	ATCTCTCAAGCGCCATGCA

### RV-1B *in vitro* infection

For H1HeLa cells, 10^5^ cells/well were seeded in a 24-well plate for 24 h prior to infection. For MYADM knockdown, cells were treated with siRNA as described above for 24 h prior to infection. Cells were washed with DPBS and incubated with RV-1B 2 × 10^4^ PFU/well for 1 h. After 1 h, cells were washed and replaced with fresh media and incubated at 35°C for 48 h. The optimal temperature for RV-1B infection in human airway epithelial cells is 35^0^ C, which was used for these studies. For AECs, at day 21 of differentiation, cells were washed with DPBS and pretreated with 75 μl of media on an apical chamber and 500 µl on a basal chamber. On day 22, the cells were washed with DPBS and incubated with RV-1B 2 × 10^4^ PFU/well for 4 h at 35°C. Then, cells were washed, replaced with fresh media, and incubated at 35°C for 48 h.

### Poly I:C stimulation

H1HeLa cells were seeded onto a 24-well plate and cultured overnight. Poly I:C (Tocris, Bristol, UK) was diluted in the media to a final concentration of 10 μg/ml and incubated with H1HeLa cells in a 24-well plate for 48 h at 37°C. Optimal poly I:C stimulation is at 37^0^ C, which was used for these studies. Cells were harvested for qRT-PCR.

### Gene transcription assay by qRT-PCR

Cells were lysed with TRIzol reagent (Invitrogen, San Diego, CA), and extracted RNAs were quantified by a NanoDrop One spectrophotometer (Thermo Fisher Scientific). cDNAs were synthesized using iScript cDNA Synthesis Kit (Bio-Rad Laboratories, Hercules, CA). Gene amplification was performed on QuantStudio 3 (Thermo Fisher Scientific) with TaqMan Universal Master Mix II (Applied Biosystems) or PowerUp SYBR Green Master Mix (Applied Biosystems) with probes and primers (Integrated DNA Technologies), as shown in **Table 2**. The target genes of interest are antiviral genes (interferon-lambda, *IFN-Λ*, interferon-beta 1, *IFN-β1*, interferon-stimulated gene 15, *ISG15*, IFN-Λ receptor 1, *IFNΛ-R1*, ubiquitin specific peptidase 18, *USP18*), inflammatory genes (interleukin-8, *IL-8*, signal transducer and activator of transcription 1, *STAT1*, *STAT3*, calcium-activated chloride channel regulator 1, *CLCA1*, C–X–C motif chemokine ligand 11, *CXCL11*), and receptors for cell signaling and rhinovirus entry (Toll-like receptor 3, *TLR3*, melanoma differentiation associated gene 5, *MDA5*, low-density lipoprotein receptor, *LDLR*, and *ICAM-1*).

### Flow cytometry assay

Cells were harvested by trypsinization for 5 min and neutralized with FBS. After washing, cells were incubated in 1:100 anti MYADM antibody (Bioss Antibodies, Woburn, MA) in 2% FBS PBS at 4°C for 30 min. Then, the cells were washed and incubated with 1:100 anti-rabbit IgG Alexa Fluor 647 (Cell Signaling Technology, Danvers, MA). Next, cells were stained with Annexin V-FITC and PI using apoptosis detection kit (BD Pharmingen, San Diego, CA) and subsequently analyzed on Attune NxT Flow Cytometer (Thermo Fisher Scientific).

### Human IL-8 ELISA

At the indicated time point, culture media were collected and kept at −80°C until further analysis. One day before the assay, a capture antibody provided by the human IL-8 ELISA kit (Invitrogen, Carlsbad CA) was coated onto a 96-well plate. The media were diluted 1:20 in an assay buffer and loaded into duplicated wells along with a detection antibody. Samples and standard IL-8 dilutions were incubated as per the manufacturer’s instruction. The absorbance at 450 nm was extrapolated into a concentration in picograms/ml using a standard IL-8 curve.

### Allergic airways disease model

The protocol was approved from the Institutional Animal Care and Use Committee (IACUC for Ledford, 15-575) at the University of Arizona (29). Briefly, 8-week-old C57BL/6 mice (Jackson Laboratories, Bar Harbor, ME) were sensitized using 50 μg ovalbumin (OVA) in alum or sterile saline intraperitoneally (i.p.) on days 0 and 7 and challenged intranasally (i.n.) on days 14, 15, and 16. On day 18, left lung lobes were collected and fixed with 10% formalin. The formalin-embedded lung sections were prepared for immunohistochemistry.

### Immunohistochemistry

After deparaffinized and antigen recovery, mouse lung sections were permeabilized, blocked, and incubated with 1:50 dilution of anti-MYADM (Bioss Antibodies) at 4°C overnight. Then, bound antibodies were captured with anti-rabbit IgG Alexa Fluor 488 (Cell Signaling Technology). Next, they were further incubated with 1:200 dilution of either anti-Ac-αTubulin (Cell Signaling Technology), anti-CYP2F2 (Santa Cruz Biotechnology, Santa Cruz, CA), anti-KRT5 (Invitrogen), anti-CC16 (Santa Cruz Biotechnology), or anti-MUC5AC (Invitrogen) for 2 h at 37°C. Bound antibodies were detected with anti-mouse IgG-Alexa Fluor 555 (Cell Signaling Technology). Images were taken using Leica DMI6000 with 40× magnification.

### Statistical analysis

All analyses were done in GraphPad Prism software and were considered statistically significant when P-value < 0.05. Gene expression, IL-8 secretion, and flow cytometry data were presented as mean± SEM from three independent experiments, and they were analyzed by one-way ANOVA and Tukey’s *post-hoc* multiple comparisons. The intensity of immunohistochemistry staining was analyzed by unpaired t-test. Differential cell count of human AECs was analyzed using two-way ANOVA. Gene transcriptions of MYADM knockdown in H1HeLa cells were analyzed by unpaired t-test. The correlation of transcription profiles was examined using Pearson correlation coefficient analysis.

## Data availability statement

The raw data supporting the conclusions of this article will be made available by the authors, without undue reservation.

## Ethics statement

The studies involving humans were approved by University of Arizona Institutional Review Board. The studies were conducted in accordance with the local legislation and institutional requirements. The human samples used in this study were acquired from primarily isolated as part of your previous study for which ethical approval was obtained. Written informed consent for participation was not required from the participants or the participants’ legal guardians/next of kin in accordance with the national legislation and institutional requirements. The animal study was approved by University of Arizona Institutional Animal Care and Use Committee. The study was conducted in accordance with the local legislation and institutional requirements.

## Author contributions

ST and AD carried out experiments; ST, MN and JL designed experiments; ST, JL carried out data analysis; FP provided human samples; MN and JL provided funding; ST wrote the paper; all authors reviewed and edited the paper. All authors contributed to the article and approved the submitted version.
