# Supplementary material for: Myeloid-associated differentiation marker is associated with type 2 asthma and is upregulated by human rhinovirus infection
Source: Front Immunol. 2023 Aug 11;14:1237683. doi: 10.3389/fimmu.2023.1237683 (PMC10450947; doi:10.3389/fimmu.2023.1237683)
Supplement: Supplementary file 1 [file DataSheet_1.docx]

Supplementary Material

**Myeloid-Associated Differentiation Marker (MYADM) is Associated with Type 2 Asthma and is Up-regulated by Human Rhinovirus Infection**

Sasipa Tanyaratsrisakul^1^, Alane Blythe C. Dy^1^, Francesca Polverino^2^, Mari Numata^3^, Julie G. Ledford^1,4*^

*** Correspondence:**Julie G. Ledford, 1230 N Cherry Avenue, BSRL Building, Tucson, AZ 85719; Phone: 520-626-0276; E-mail: jledford@arizona.edu


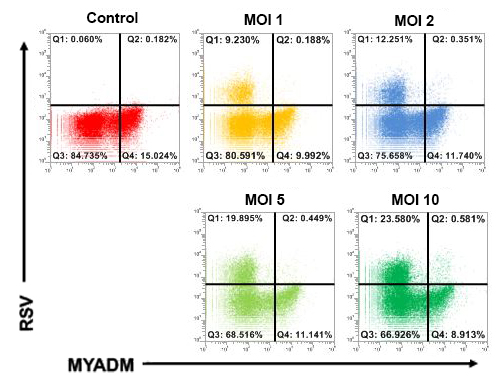


**Supplementary Figure 1. RSV infection did not increase MYADM expression in H1HeLa cells.** H1HeLa cells were infected with RSV encoding green fluorescent protein at MOI of 1, 2, 5, and 10 and subsequently incubated at 37°C for 24 hrs. Cells were stained with anti MYADM antibody followed by anti-rabbit IgG conjugated with Alexa Fluor 647. Dot plot shows the representative result from flow cytometry assay with red fluorescent (MYADM) on X axis and green fluorescent (RSV) on Y axis.

**
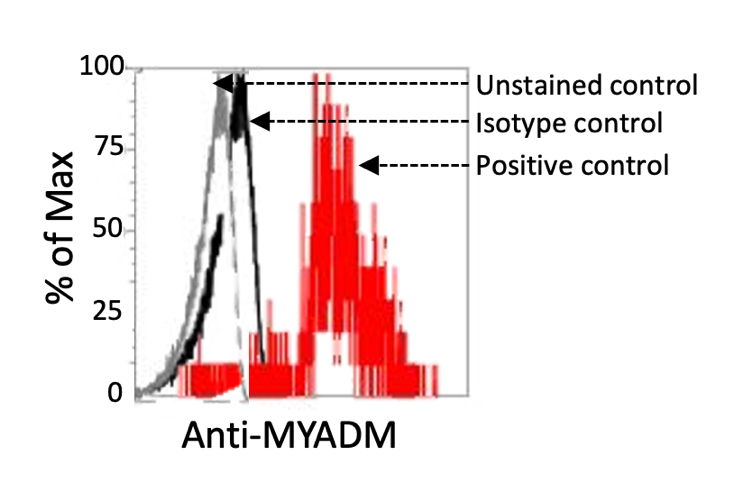
**

**Supplementary Figure 2.** Assessment of MYADM antibody by flow cytometry as compared to unstained and isotype controls in HeLa cells. Positive control indicates that cells have been challenged with RV1B prior to staining.

**A**

**
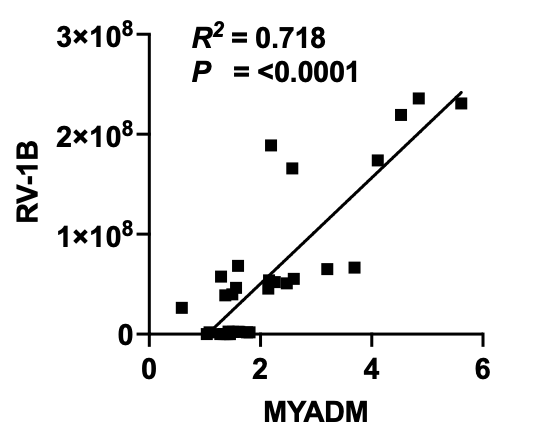

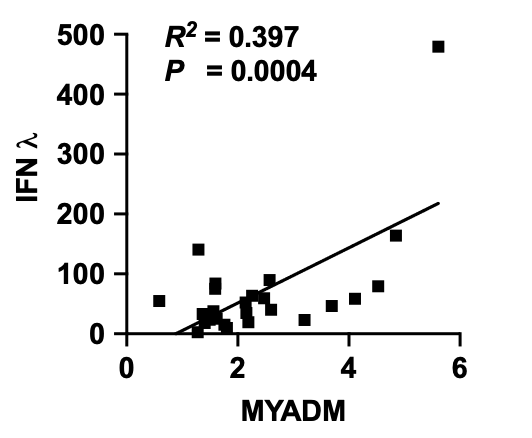

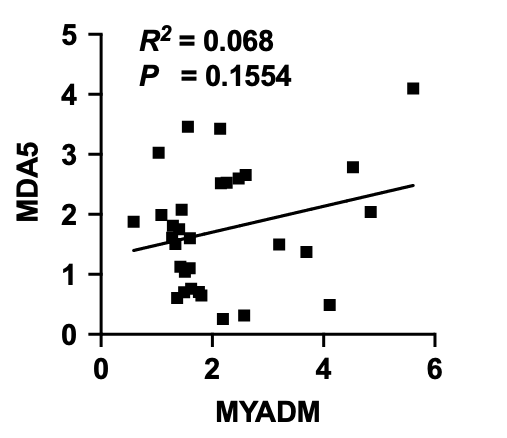

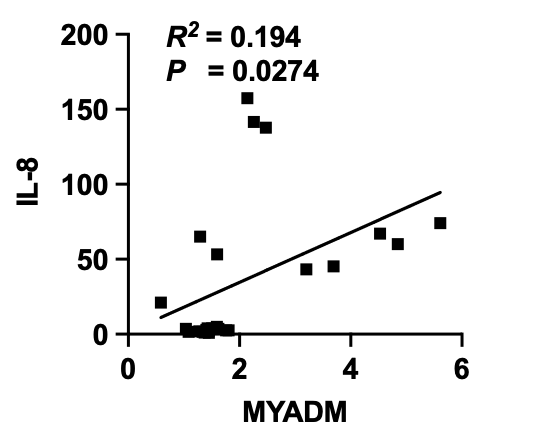

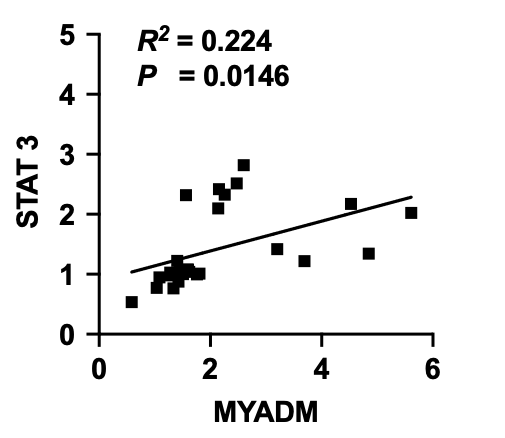

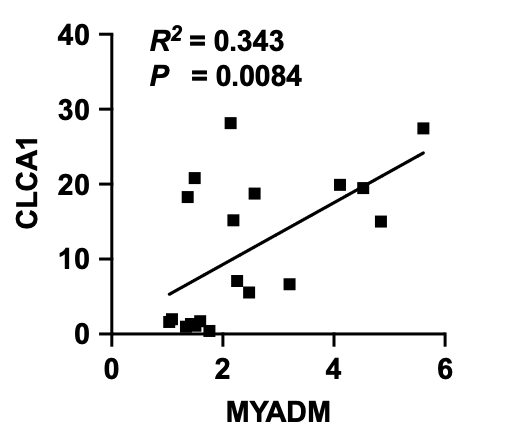
**

**B**

**
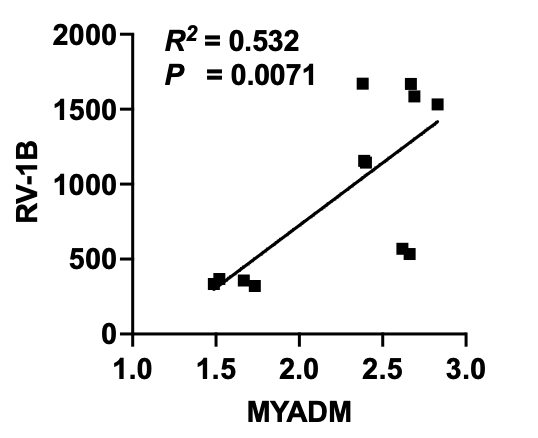

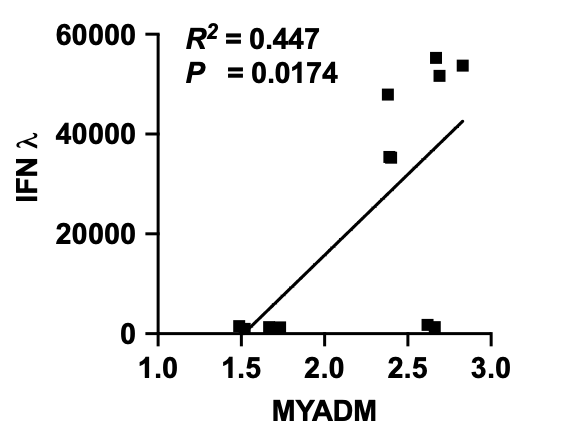
****** **
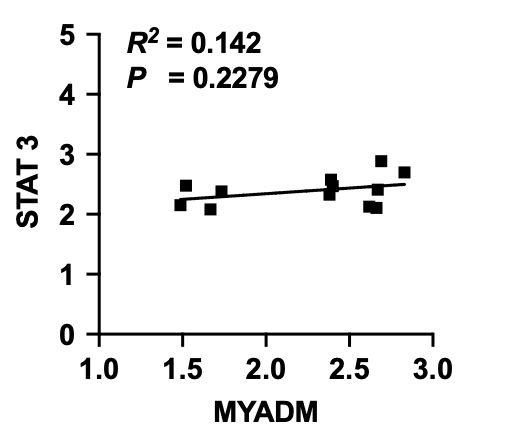
**
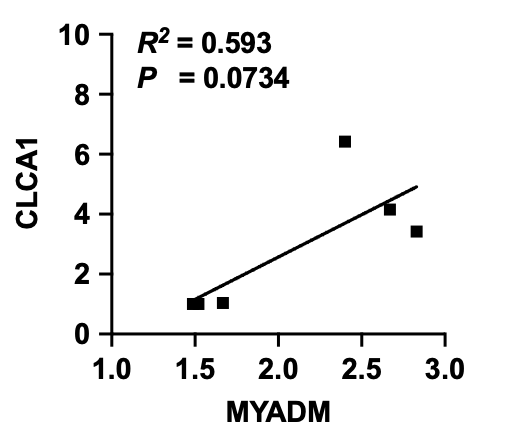

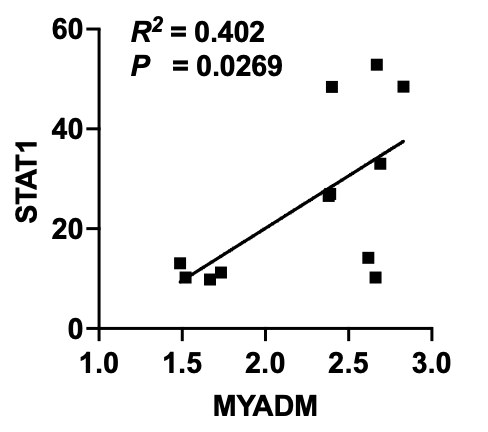

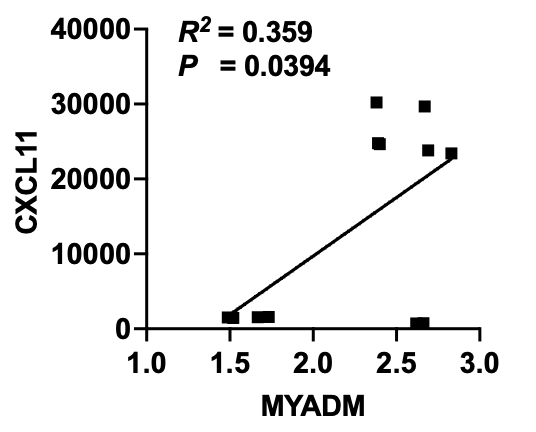

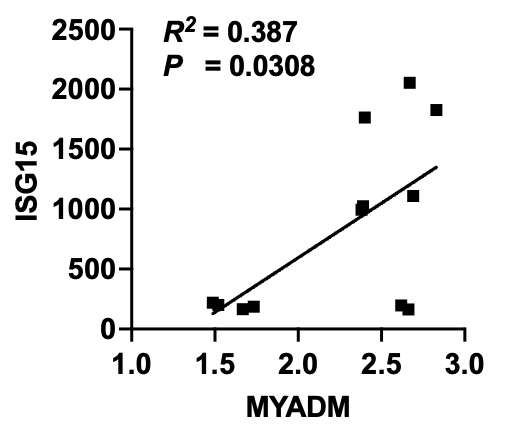

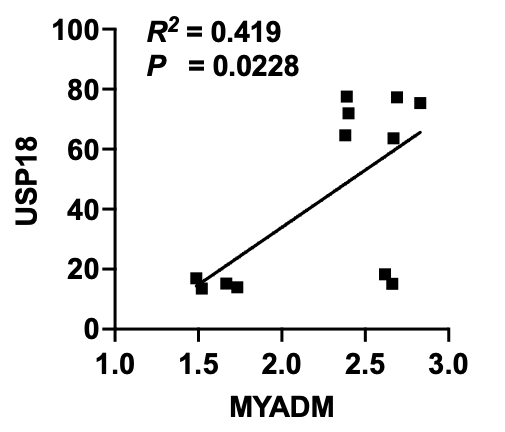

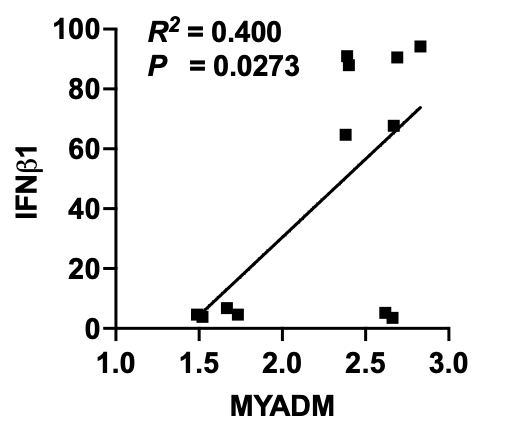

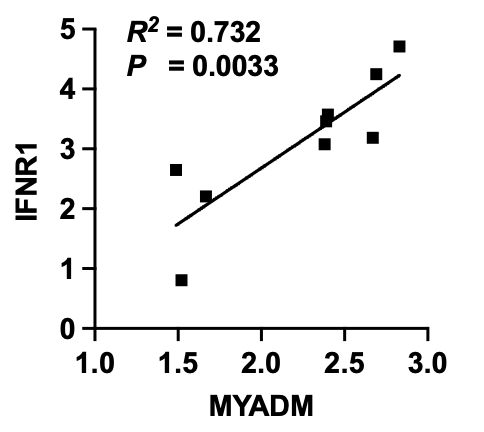

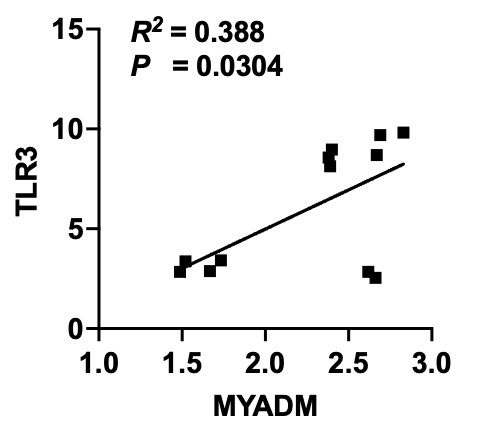

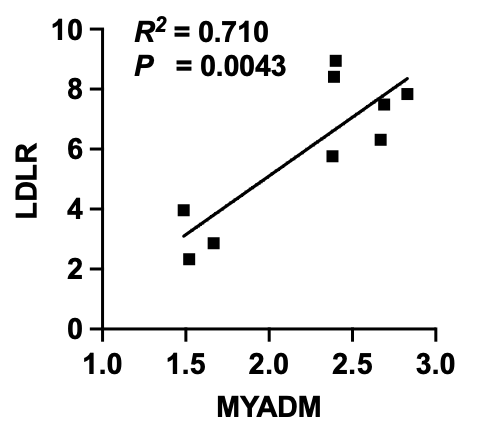

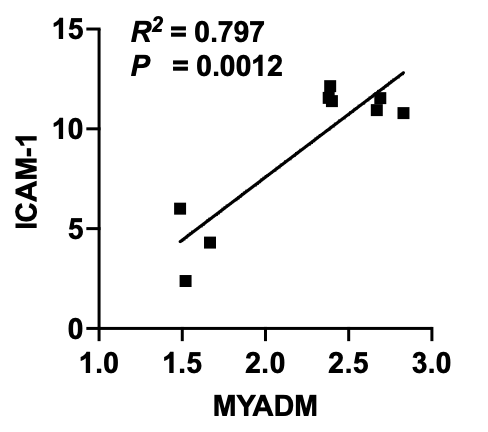


**Supplementary Figure 3. MYADM expression correlated with RV-1B replication and RV-1B-induced responses.** H1HeLa cells (**A**) or differentiated AECs grown at ALI (**B**) were infected with RV-1B at MOI 0.1 for 48 hr. Transcription of target genes were quantified by qRT-PCR. The fold changes to control normalized to GAPDH from 3 independent experiments were plotted on the graph. Correlation of genes expression compared to MYADM was calculated using Pearson correlation coefficients in GraphPad Prism software.
